# A Method for Calculating the Area of *Zostera marina* Leaves from Digital Images with Noise Induced by Humidity Content

**DOI:** 10.1155/2014/786896

**Published:** 2014-05-04

**Authors:** Cecilia Leal-Ramirez, Hector Echavarria-Heras

**Affiliations:** Centro de Investigación Científica y Educación Superior de Ensenada, Carretera Ensenada-Tijuana No. 3918, Zona Playitas, 22860 Ensenada, BC, Mexico

## Abstract

Despite the ecological importance of eelgrass, nowadays anthropogenic influences have produced deleterious effects in many meadows worldwide. Transplantation plots are commonly used as a feasible remediation scheme. The characterization of eelgrass biomass and its dynamics is an important input for the assessment of the overall status of both natural and transplanted populations. Particularly, in restoration plots it is desirable to obtain nondestructive assessments of these variables. Allometric models allow the expression of above ground biomass and productivity of eelgrass in terms of leaf area, which provides cost effective and nondestructive assessments. Leaf area in eelgrass can be conveniently obtained by the product of associated length and width. Although these variables can be directly measured on most sampled leaves, digital image methods could be adapted in order to simplify measurements. Nonetheless, since width to length ratios in eelgrass leaves could be even negligible, noise induced by leaf humidity content could produce misidentification of pixels along the peripheral contour of leaves images. In this paper, we present a procedure aimed to produce consistent estimations of eelgrass leaf area in the presence of the aforementioned noise effects. Our results show that digital image procedures can provide reliable, nondestructive estimations of eelgrass leaf area.

## 1. Introduction

*Zostera marina* also known as eelgrass is a relevant seagrass species, which supplies significant amounts of organic materials to food webs in shallow coastal environments and provides habitat (in bays, lagoons, or estuaries) for many fishes and their larvae [1]. Eelgrass beds can also help remediate contaminated sediments [2], filter and retain nutrients from the water column [3], help in the stabilization of sediments [4], and reduce erosion forces by stumping wave energy, thus promoting the stabilization of adjacent shorelines [5]. However, the permanence of eelgrass beds—as well as those formed by other seagrass species—is currently being threatened by anthropogenic influences to such an extent that special conservation efforts are needed [6]. This requires the development of accurate and cost-effective procedures aimed at obtaining scientific knowledge about the pertinent growth dynamics. This is particularly relevant in assessments of restoration projects, where the use of noninvasive data gathering techniques turns out to be of fundamental importance.

The characterization of eelgrass biomass and its dynamics is an important input for the assessment of the overall status of both natural and transplanted eelgrass populations. In eelgrass the basic unit for studying biomass and its production is the shoot, which includes sheaths, leaves, rhizomes, and roots. Biomass consists of an aboveground component formed by sheaths and leaves and a belowground constituent formed by rhizomes and roots. Root emergence occurs at leaf scars, also known as rhizome nodes. The production of leaves and rhizome nodes is connected such that each leaf produced is linked to a rhizome node. Hence, the overall production of shoots can be estimated by measuring the production of leaves [7]; this makes us know the growth rate of leaves fundamental to the assessment of eelgrass populations [8]. Moreover, estimations of leaf biomass and leaf-growth rates are keys to assessing the reestablishment of ecological functioning in restored areas. Nevertheless, traditional methods for the estimation of eelgrass leaf biomass and the related leaf growth rates are destructive and time consuming. Even though these procedures do not damage natural seagrass populations, they could produce undesirable effects on transplant experiments. Favorably, the conspicuous growth form of eelgrass makes it possible to introduce proxies that allow assessments while avoiding invasive interference. Moreover, estimations of leaf biomass or productivity in eelgrass can be efficiently obtained using allometric alternatives, which state these variables in terms of leaf length or area [9, 10]. But even though leaf architecture in eelgrass makes length a consistent descriptor of area, allometric models that express leaf biomass in terms of linked area perform relatively better than those involving leaf length as an independent variable. Therefore, for consistent allometric estimations of leaf biomass or productivity of eelgrass it is convenient to produce reliable estimations of leaf area. The observed ribbon-like appearance of the leaves in* Zostera marina* is a feature that permits obtaining direct and fairly accurate estimations of blade length *l* and width *h*. These variables provide convenient estimations of the corresponding blade area *a*, through the leaf length times width proxy [1]. If we used the symbol *o* as a subscript to represent observed values for the above named variables, then estimations of leaf area obtained through this proxy are given by

(1) $$\begin{array}{c} a_{o}=l_{o}\cdot h_{o} \end{array}$$

which combined with allometric methods could simplify assessments of eelgrass leaf biomass and productivity [9, 10].

Digital image processing techniques were initially aimed to calculate the area of leaves for terrestrial plants [11–13]. These methods provide simplified estimations of biologically relevant variables. For example, Patil and Bodh [14] used area of* sugarcane* leaves for plant growth monitoring to analyze manure scarcity and environmental stress and to assess disease severity. Lü et al. [15] used leaf area measurement to assess long-term influences on yield and because it is a fundamental index in crop growth and nurturing practice. Although, leaf area in eelgrass can be conveniently obtained by means of (1), and both *l*_*o*_ and *h*_*o*_ can be directly measured on most sampled leaves, methods based on digital image processing could be adapted in order to simplify these tasks. Moreover, eelgrass leaf area can be directly estimated from digital imagery by using the Monte Carlo method [1]: if we let *a*_*mc*_ denote these estimations, then they are obtained through

(2) $$\begin{array}{c} a_{mc}=\frac{\text{LPN}}{{\text{UPN}}^{2}}, \end{array}$$

where LPN is the number of points placed inside the considered leaf area and UPN^2^ stands for the number of points contained in a unit area.

Besides, the Monte Carlo method eelgrass leaf area could be also obtained from digital images by using the length times width proxy of (1). Indeed, if *l*_*d*_ and *h*_*d*_, respectively, denote leaf length and width obtained from the associated digital image, then these variables can be estimated through

(3) $$\begin{array}{c} l_{d}=\frac{{\text{np}}_{l}}{\text{unp}}, \end{array}$$

(4) $$\begin{array}{c} h_{d}=\frac{{\text{np}}_{h}}{\text{unp}}, \end{array}$$

where np_*l*_ and np_*h*_ are, respectively, obtained by counting the number of points contained over the length and width dimensions of the leaf, and unp is the number of points contained in the appropriate distance measurement unit. Therefore, denoting by means of *a*_*d*_ the associated leaf area, we will correspondingly have

(5) $$\begin{array}{c} a_{d}=l_{d}\cdot h_{d}. \end{array}$$

Nevertheless, when using either (2) or (5) to produce estimations of leaf area we must be aware that some* Zostera marina* leaves could be very long or present curvatures, among irregularities caused by environmental factors like grazing or drag forces. The influence of these factors could affect image quality, which could produce biased estimations for leaf area. These effects have been partially addressed by Ramfos et al. [16], who proposed a method based on image processing techniques for measurements of a* Zostera marina* leaf by taking into account the effects of curvature on accuracy. Yet another important factor which we address here concerns the effects that the humidity contents of a leaf can originate in image processing. In fact, once leaves are removed from a shoot they begin to lose water and degrade. Hence, if leaves cannot be processed immediately after being collected, it is important to keep them in a manner that reduces changes in shape [17]. Therefore, an efficient digitalizing of a* Zostera marina* blade requires maintenance of an optimal humidity for increased image fidelity. On the other hand, humidity contents in a leaf can induce noise to an image by adding extraneous information, which usually manifests by pixel value misidentification.

Data published by Echavarria-Heras et al. [1], taken over a comprehensive sampling experiment, show that measured maximum width for a* Zostera marina* leaf is 6 mm. Surely, other authors report a variation range from 1.5 to 12 mm for this estimation [18]. A wide variation range in width in conjunction with noise due to humidity content can increase uncertainty in blade width measurements obtained from digitalized leaf images. This makes it necessary to devise a way that allows discriminating the concomitant error spreading over leaf area assessments. So far, an approach that integrates among others techniques, one aimed to handle noise effects induced by the humidity contents on a* Zostera marina* leaf, has not been produced. In this study, we conceived a method which using criteria based on statistical analysis techniques reduces the effects that noise linked to the humidity contents of a* Zostera marina* leaf produces on the accuracy of associated area estimations obtained from a digital image.

## 2. Conceptual Framework for Image Processing 

Our arrangement depends in a fundamental way on the concept of the peripheral or bordering contour of a bidimensional enclosure or domain. Several definitions of peripheral contour exist, being each one appropriate for different settings. Our interpretation is similar to the perimeter definition of a regular pattern in geometry. More accurately, a peripheral contour in the present settings will be defined as the sequence of boundary pixels of a digitalized eelgrass leaf. Moreover, for a reasonable identification of the area of the pertinent blade it is imperative that in the extents of the corresponding image minimal changes of color levels occur, even though around its outer contour abrupt changes of a color levels could be shown. The effect projected by humidity adds to leaf area pixels placed between the pixels captured by the digital image of the leaf itself and others belonging to its background. Hence, a reliable imbedding of the area of a leaf into an image requires the unambiguous identification of the pixels on its surrounding contour.

Our design is aimed to the aforesaid identification in the presence of noise due to the humidity content in the leaf. For the incumbent characterization, our system uses a quantitative setup developed on the basis of the concepts of adjacency, vicinity, connectivity, and tolerance of similarity between pixels. We briefly describe these notions in what follows.

Two pixels are adjacent if and only if they share one of their borders, or at least one of their corners. Two pixels are neighbors if they fulfill the definition of adjacency. Formally, the vicinity *V*_*p*_(*x*, *y*) of the point *P*(*x*, *y*) is defined through

(6) Vp(x,y)=(x+1,y),(x−1,y),(x,y+1),(x,y−1),(x+1,y+1),(x+1,y−1),(x−1,y+1),(x−1,y−1).

Without loss of generality, we explain the notion of tolerance of similarity by referring to the RGB format description of a color. This allows quantifying tonality in terms of the intensities of the constituting primary colors: red, green, and blue. To indicate at which amount each one of these colors is mixed to produce a given tonality a value is assigned to each prime color; for example, the value 0 means that a given basic color does not appear in the mix, but if a chief color component is nonvanishing it means that it contributes to the mix in a given intensity. We set *C*_max⁡_ which identifies the colors number to be used through the whole image processing task; for an RGB color space we have *C*_max⁡_ = 256. Usually, the intensity of each of the primary colors appearing in a mix is measured on a scale ranging from 0 to *C*_max⁡_ − 1. The set of all color intensities can be represented in the form of a cube in the cartesian coordinate system, where each color is a point on the surface or in its interior. Given points *P* = (*p*_1_, *p*_2_,…, *p*_*n*_) and *Q* = (*q*_1_, *q*_2_,…, *q*_*n*_) in an RGB color space, we will define the distance *d*_*E*_(*P*, *Q*) between them through

(7) $$\begin{array}{c} d_{E}\left(P,Q\right)=\sqrt{\overset{n}{\underset{i=1}{\sum }}{\left(p_{n}-q_{n}\right)}^{2}}. \end{array}$$

Moreover, given a point *P* in an RGB color space, a second one *Q* with the greatest similarity to *P* is the one placed at the smallest distance *d*_*E*_(*P*, *Q*). Furthermore, let ST(*x*) = [0, *x*] be a color tonality range, with *x* being the number of different colors included. Then, we must have 1 ≤ *x* ≤ *C*_max⁡_ − 1 and we will say that two pixels *P* and *Q* are similar to a tolerance limit ST(*x*) if the inequality

(8) $$\begin{array}{c} d_{E}\left(P,Q\right)\leq x \end{array}$$

is satisfied. In what follows the range ST(*x*) will be simply called “tolerance of similarity” and the upper bound *x* can be interpreted as the maximum distance that two points located within the extent of an object can attain in an RGB color space in order to be considered similar. Connectivity between pixels is used to identify the limits in objects and regions in an image. We will say that two pixels *P* and *Q* are connected with tolerance of similarity ST(*x*) if they fulfill the definition of adjacency and also if inequality (8) holds.

## 3. The Image Selection Method

The procedure to obtain efficient estimations of *l*_*d*_, *h*_*d*_, and *a*_*mc*_ requires two stages. On a first one we create a digital image for each one of the collected leaves. Then, we set *C*_max⁡_ and continue by choosing an interval of tolerance of similarity ST(*x*) with 0 ≤ *x* ≤ *C*_max⁡_ − 1; we use this to obtain the peripheral contour of each one of the available leaf images and from them the linked *l*_*d*_, *h*_*d*_, and *a*_*mc*_ values. Different intervals ST(*x*) will produce different estimations for *l*_*d*_, *h*_*d*_, and *a*_*mc*_, and consequently we must rely on a criterion for the selection of the ST(*x*) range that produces the most accurate estimations. To carry out this task in a second stage of the method we arrange leaf length data into groups of leaves whose size differences are bounded by a preferred tolerance *q* and use that arrangement to obtain related statistics *β*_*a*_ and *λ*_*a*_ that are used to implement what we call the IS_*x*_ selection index. In what follows we describe pseudo-codes for the above referred stages. Detailed formulae are presented in the appendices. Tables 1, 2, 3, 4, and 5 summarize the involved notation.

### 3.1. The Procedure to Obtain *l*_*d*_, *h*_*d*_, and *a*_*mc*_ Assessments

(a.1) Choose a color format and set *C*_max⁡_.
(a.2) Load the leaf image.
(a.3) Enter an interval of tolerance of similarity ST(*x*); 1 ≤ *x* ≤ *C*_max⁡_ − 1.
(a.4) Select a starting point inside the loaded leaf image.
(a.5) Find the contour of the leaf image through (6), (7), and (8) (these equations identify all adjacent pixels falling within the selected interval of tolerance of similarity ST(*x*)).
(a.6) Obtain *l*_*d*_, *h*_*d*_, and *a*_*d*_ by using (3), (4), and (5), respectively.
(a.7) Obtain *a*_*mc*_ by using (2).
(a.8) Record ST(*x*) and the associated *l*_*d*_, *h*_*d*_, *a*_*d*_, and *a*_*mc*_ estimations.
(a.9) Repeat steps 2–8 for each one of the available leaf images.
(a.10) Change the ST(*x*) interval and jump to step (a.3).

Different ST(*x*) intervals will produce through the above procedure different estimations for *l*_*d*_, *h*_*d*_, *a*_*d*_, and *a*_*mc*_. We now outline a procedure for the selection of the image that produces the most accurate estimations *a*_*d*_ or *a*_*mc*_ for the observed leaves area *a*_*o*_. This requires the identification of the interval of tolerance of similarity ST(*x*) that yields the smallest values of the selection index IS_*x*_ defined by (9) below.

### 3.2. The Method for the Selection of an Optimal *ST*(*x*) Interval

(b.1) For the entered ST(*x*) interval, use ((E.9)) to calculate *λ*_*a*_ (this value gives the proportion of leaves for which *a*_*d*_ produces consistent estimations of *a*_*o*_).
(b.2) For the entered ST(*x*) interval, use ((E.10)) to calculate *β*_*a*_ (this value yields the proportion of leaves for which *a*_*d*_ overestimates observed leaf area *a*_*o*_).
(b.3) For the entered ST(*x*) interval, calculate the value of the image selection index IS_*x*_ according to
  (9) $$\begin{array}{c} {\text{IS}}_{x}=\frac{\beta_{a}}{\lambda_{a}}. \end{array}$$
(b.4) Record both ST(*x*) and IS_*x*_.
(b.5) Change the ST(*x*) interval and repeat steps (b.1) to (b.3) until all the ST(*x*) intervals generated in Section 3.1 are exhausted.
(b.6) Choose the ST(*x*) interval that produces the smallest value of IS_*x*_ for image processing and leaf area *a*_*d*_ estimations.

The above selection index IS_*x*_ criterion can be adapted for Monte Carlo method estimations of leaf area. It becomes

(10) $$\begin{array}{c} {\text{IS}}_{xmc}=\frac{\beta_{amc}}{\lambda_{amc}}, \end{array}$$

where *λ*_*amc*_ and *β*_*amc*_ are, respectively, given by ((E.11)) and ((E.12)) in Appendix E and are equivalent to *λ*_*a*_ and *β*_*a*_ correspondingly.

## 4. Results

### 4.1. Leaf Data Grouping

The present data set was obtained by randomly sampling 5 shoots biweekly from January through December 2009 in a* Zostera marina* field at Punta Banda estuary, a shallow coastal lagoon located near Ensenada, Baja California, Mexico (31° 43–46 N and 116° 37–40 W). For each sampled leaf, a millimeter ruler was used to obtain *l*_*o*_ to the nearest 1/10 mm taken as the distance from the top of the sheath to the leaf tip. Meanwhile, *h*_*o*_ was measured at a point halfway between the top of the sheath and the tip. Observed leaf area estimations *a*_*o*_ were calculated by means of (1).

We obtained *l*_max⁡_ = 460 mm. For data grouping we selected *n* = 46, so we acquired *q* = 10 mm and for the interval [0, *l*_max⁡_] we formed a partition *P*_0_^460^ of disjoint intervals *I*_*k*_ of the form *I*_*k*_ = {*l* | *q*(*k* − 1) ≤ *l* < *qk*}, with 1 ≤ *k* ≤ 46. Hence, as described in the appendices for each value of the index *k*, we formed a group *G*_*k*_(*l*) containing leaves with sizes varying in the interval *I*_*k*_ (Table 6). Longer and older leaves displayed darker tonalities than younger and shorter ones. Moreover, leaves with lengths varying on a given partition interval *I*_*k*_ displayed a similar color distribution. For some of the partition intervals there was at most one leaf with length placed in the linked variation range. Therefore, these groups are not taken into account because they do not provide information for the statistical analysis (see bold in Table 6).

### 4.2. Image Selection Procedure

For each one of the leaves in the collection *C*_*G*_ of groups *G*_*k*_(*l*), we applied the procedure described in the pseudo-code (a) aimed to detect the points on the associated peripheral contour and to get the concomitant *l*_*d*_, *h*_*d*_, *a*_*d*_, and *a*_*mc*_ estimations. For that purpose we used a variety of equivalence of tones, which permitted an unambiguous framing of the extent of the leaf. A RGB 256 color format was used for all leaves images. Hence, we set *C*_max⁡_ = 256. Therefore, different tolerances of similarity ranges, ST(*x*) = [0, *x*] with 1 ≤ *x* ≤ 255, were used. Moreover, the procedure was automatically applied up to 256 times on each individual leaf image. For every tolerance of similarity interval ST(*x*) we selected a starting point inside a chosen leaf image and we identified all adjacent pixels falling within the named similarity range. This identified the peripheral contour of the leaf image so the linked *l*_*dj*_^*k*^, *h*_*dj*_^*k*^, and *a*_*dj*_^*k*^ assessments as well as leaf area estimations *a*_*mcj*_^*k*^ were acquired (see B.3 in Appendix B).

**Figure 1:**
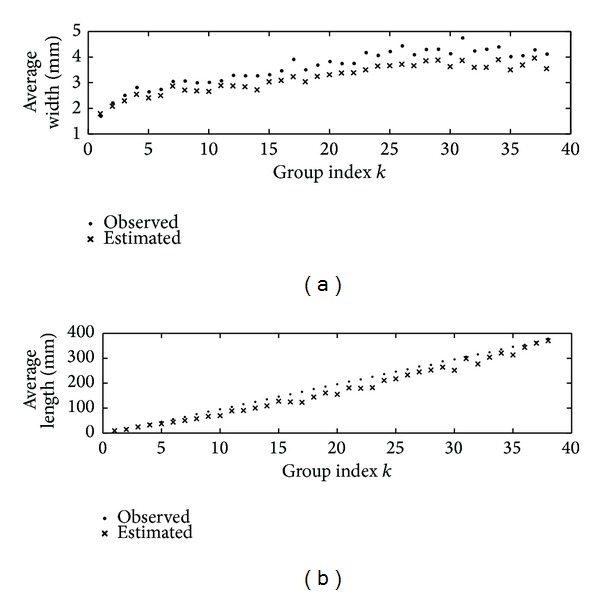
(a) Comparison of observed ${\overset{-}{h}}_{o}^{k}$ and image estimated ${\overset{-}{h}}_{d}^{k}$ width averages taken over groups *G*_*k*_(*l*). (b) Comparison of observed ${\overset{-}{l}}_{o}^{k}$ and image estimated ${\overset{-}{l}}_{d}^{k}$ length averages taken over groups *G*_*k*_(*l*) (see Table 6). The values obtained from digitized leaves were estimated by using ST(68).

**Figure 2:**
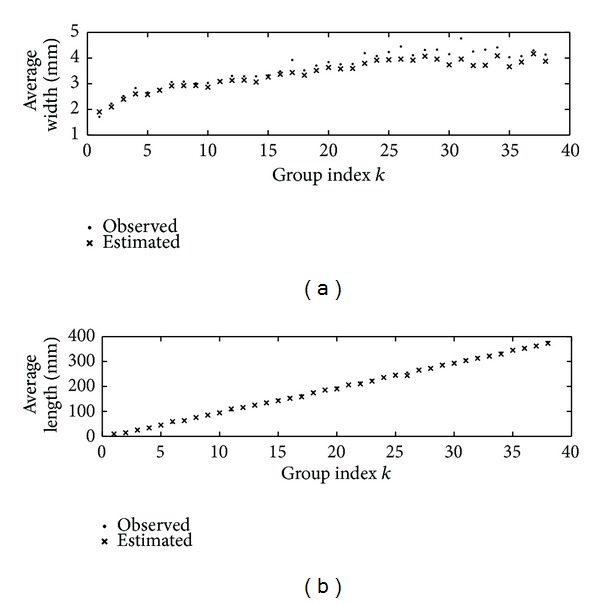
(a) Comparison of observed ${\overset{-}{h}}_{o}^{k}$ and image estimated ${\overset{-}{h}}_{d}^{k}$ width averages taken over groups *G*_*k*_(*l*). (b) Comparison of observed ${\overset{-}{l}}_{o}^{k}$ and image estimated ${\overset{-}{l}}_{d}^{k}$ length averages taken over groups *G*_*k*_(*l*) (see Table 6). The values obtained from digitized leaves were estimated by using ST(68). The values obtained from digitized leaves were estimated by using ST(128).

**Figure 3:**
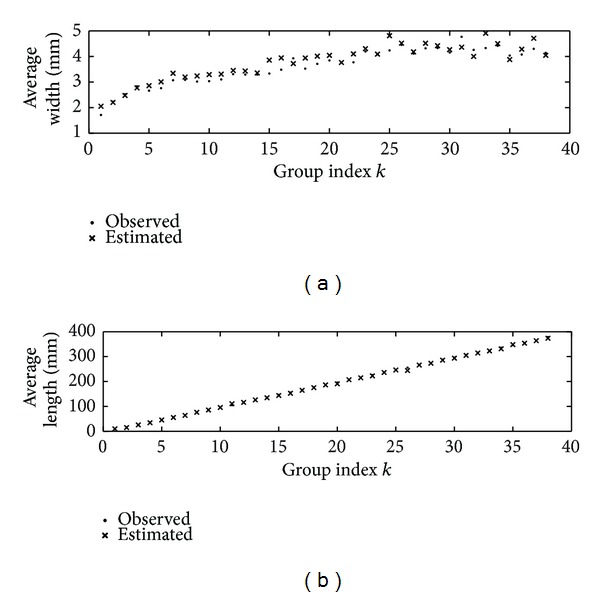
(a) Comparison of observed ${\overset{-}{h}}_{o}^{k}$ and image estimated ${\overset{-}{h}}_{d}^{k}$ width averages taken over groups *G*_*k*_(*l*). (b) Comparison of observed ${\overset{-}{l}}_{o}^{k}$ and image estimated ${\overset{-}{l}}_{d}^{k}$ length averages taken over groups *G*_*k*_(*l*) (see Table 6). The values obtained from digitized leaves were estimated by using ST(68). The values obtained from digitized leaves were estimated by using ST(192).

For each group *G*_*k*_(*l*) of leaves determined by the partition *P*_0_^460^ we calculated deviation values *δ*_*h*_^*k*^ and *δ*_*l*_^*k*^ and their averages ${\overset{-}{\delta }}_{h}$ and ${\overset{-}{\delta }}_{l}$ taken over *C*_*G*_. We also calculated the associated standard deviation values *σ*_*δh*_ and *σ*_*δl*_ (see Appendix D) and the proportions, *λ*_*h*_, *λ*_*l*_. *θ*_*h*_, *θ*_*l*_, *λ*_*a*_ *β*_*a*_, *λ*_*amc*_, and *β*_*amc*_ (see Appendix E); calculated values are presented in Tables 7 and 8. Values of the image selection index IS_*x*_ were obtained and compared. For easy of presentation we focus on the results obtained for ST(68), ST(128), and ST(192) which include the smallest obtained value for IS_*x*_ (Table 8). Figures 1, 2, and 3 display comparisons of the averages ${\overset{-}{l}}_{o}^{k}$ and ${\overset{-}{h}}_{o}^{k}$ of observed leaf lengths and widths in groups *G*_*k*_(*l*) versus estimations ${\overset{-}{l}}_{d}^{k}$ and ${\overset{-}{h}}_{d}^{k}$ from images acquired using these tolerance of similarity intervals.

For 1 ≤ *x* ≤ 67, *λ*_*l*_ and *λ*_*h*_ values were greater than those calculated for ST(68). Moreover, *λ*_*l*_ and *λ*_*h*_ values obtained using 1 ≤ *x* ≤ 127 were greater than those obtained using ST(128) but smaller than those obtained for 1 ≤ *x* ≤ 67. Nevertheless, *λ*_*l*_ values produced for 1 ≤ *x* ≤ 196 were smaller than those linked to 1 ≤ *x* ≤ 128, but generally *λ*_*h*_ values increased implying a greater concentration of a particular tonality within the range of colors forming the color spectrum of the image. That is, ST(*x*) broadens or reduces the collection of colors which can be taken into account for pixel identification within the extent of the image. Whenever *x* stands for a greater amount of colors relative to the assortment defining the image, the pixel selection procedure will lead to subjective identification. This influences ${\overset{-}{\delta }}_{h}$ and ${\overset{-}{\delta }}_{l}$ values in a direct way. Indeed for ST(192), ${\overset{-}{\delta }}_{h}$ was negative (Table 7) which means that most *h*_*dj*_^*k*^ values were greater than *h*_*oj*_^*k*^ values. Nevertheless, this outcome is limited by the maximum value that *x* can attain and also by image size. For 1 ≤ *x* ≤ 255, the value of *λ*_*l*_ remained unchanged.

By using the proportion values *λ*_*a*_, *β*_*a*_, *λ*_*amc*_ and *β*_*amc*_, we assessed that for ST(192) leaf area was overestimated by the method. And although for ST(128) the method slightly overestimated leaf area, for ST(*x*) intervals beyond ST(128) leaf area overestimation always increased. Moreover, in Table 9 we provide root-mean-square deviation (RMSD) values for comparisons of directly versus image obtained estimations when using ST(*x*) intervals. We can assess that a ST(128) interval produced the highest RMSD values for the comparison of directly versus image obtained estimations of leaf width, length or area. This is consistent with the smallest value obtained for the IS_*x*_ selection index. Therefore, we suggest that a similarity ST(128) interval must be set to process the present* Zostera marina *leaf image set through the method proposed.

## 5. Discussion

Deleterious effects derived from anthropogenic influences are currently increasing worldwide, threatening the health of many eelgrass meadows [19]. Remediation efforts have included transplant projects [20] and the valuation of their status, requires the estimations of key variables such as standing stock or productivity. Although shoot removal for the measurement of these variables does not damage natural seagrass populations, these procedures could produce undesirable effects on transplants. Therefore, when standing stock or productivity assessment are performed over the early stages of an eelgrass restoration experiment, data gathering approaches that avoid disruptive interference are essential. Allometric methods can provide convenient proxies, which reduce leaf biomass and growth assessments to simple blade length or area measurements. What is more, if the estimation of these leaf attributes can be done without removing the blades allometric approaches could furnish truly non-destructive assessments. Modern electronic scanning technologies could be used under water in order to produce reliable images of marine plants leaves, which guarantee non-destructive sampling of leaves length, width or area. However, insitu scanning of eelgrass leaves could add extraneous information mainly due to the inherent humidity content or to materials attached to blades like mud related particles. Hence, for estimating leaf attributes such as length or area, we must take into account that image noise effects could render biased assessments. Moreover, if we strive to use (6), the digital image counterpart of (1), we must take into account that a* Zostera marina* leaf does not show a perfect rectangular shape. We should also notice that since width attains small values, noise produced by humidity could increase the width of the digital leaf in a noticeable way which could certainly heighten uncertainty in leaf area estimations produced through (5). What is more, even when leaf area is estimated from images using Monte Carlo methods, noise effects could produce ambiguity in peripheral contour identification rendering biased results.

In the other hand, we need to be aware that since the power functions involved in allometric approaches are highly sensitive to parameter uncertainty we require consistent estimations of blade length or area [10]. Therefore, when we estimate eelgrass leaf area, using digital imagery in combination with allometric proxies, we must be aware that image noise could certainly reduce the accuracy of estimates. This makes it necessary to rely on efficient image selection methodologies for uncertainty reduction. Our results show that the present methods produce reliable results. This conclusion is mainly substantiated by the obtained values for the RMSD. We used these statistics to determine consistency between directly obtained measurements and image estimated assessments. Table 9 shows that the highest RMSD values corresponded with the smallest values obtained for the IS_*x*_selection index. Moreover, the ST(*x*) interval selected using the IS_*x*_ criteria produced also the highest RMSD values for the comparison of observed values and those obtained by means of Monte Carlo method. This justifies our claim that the proposed procedure abridged by the selection index IS_*x*_ can be expected to produce consistent estimations of the leaf attributes necessary for allometric estimation of relevant variables required to assess the status of an eelgrass population. Moreover, the presented procedure could be straightforwardly applied to other eelgrass populations or seagrass species that exhibit similar leaf architectures making leaf length times width a reliable proxy for the pertinent area.

## Figures and Tables

**Table 1:** Different symbols used in the digital image processing method.

Symbol	Description
*l*	Leaf length
*h*	Leaf width
*C*_max⁡_	Number of colors in a format of a digital image
ST(*x*) = [0, *x*]	Interval of Tolerance of Similarity
*l*_max⁡_	Maximum observed leaf length
$q=\frac{l_{\max}}{n}$	Norm of the partition for the interval [0, *l*_max⁡_]
*I*_*k*_	Partition interval of the form [*q*(*k* − 1), *qk*) for 0 ≤ *k* ≤ *n*
$P_{0}^{l_{\max}}=\overset{n}{\underset{1}{⋃}}\left(I_{k}\right)$	Collection of *n* intervals *I*_*k*_ that cover [0, *l*_max⁡_]
*G*_*k*_(*l*)	Group of leaves whose lenghts (*l*) lie in *I*_*k*_
*n*_*k*_	Number of leaves in the group *G*_*k*_(*l*)
$C_{G}=\overset{n}{\underset{1}{⋃}}G_{k}(l)$	Collection of all groups *G*_*k*_(*l*) of leaves

**Table 2:** Symbols for observed, digitally obtained variables and related averages.

Description	Observed data	Digital data	Monte Carlo data
Leaf length (*l*)	*l*_*o*_	*l*_*d*_	—
Leaf width (*h*)	*h*_*o*_	*h*_*d*_	—
Leaf area (*a*)	*a*_*o*_	*a*_*d*_	*a*_*mc*_
Length of the *j*th leaf in group *G*_*k*_(*l*)	*l*_*oj*_^*k*^	*l*_*dj*_^*k*^	—
Width of the *j*th leaf in group *G*_*k*_(*l*)	*h*_*oj*_^*k*^	*h*_*dj*_^*k*^	—
Area of the *j*th leaf in group *G*_*k*_(*l*)	*a*_*oj*_^*k*^	*a*_*dj*_^*k*^	*a*_*mcj*_^*k*^
Average length of the leaves in group *G*_*k*_(*l*)	${\overset{-}{l}}_{o}^{k}$	${\overset{-}{l}}_{d}^{k}$	—
Average width of the leaves in group *G*_*k*_(*l*)	${\overset{-}{h}}_{o}^{k}$	${\overset{-}{h}}_{d}^{k}$	—
Average area of the leaves in group *G*_*k*_(*l*)	${\overset{-}{a}}_{o}^{k}$	${\overset{-}{a}}_{d}^{k}$	${\overset{-}{a}}_{mc}^{k}$

**Table 3:** Approximation errors.

Symbols	Formal expression	Description
*e*_*lj*_^*k*^	*l*_*oj*_^*k*^ − *l*_*dj*_^*k*^	Difference of observed and image obtained leaf lengths in group *G*_*k*_(*l*).
*e*_*hj*_^*k*^	*h*_*oj*_^*k*^ − *h*_*dj*_^*k*^	Difference of observed and image obtained leaf widths in group *G*_*k*_(*l*).
*e*_*aj*_^*k*^	*a*_*oj*_^*k*^ − *a*_*dj*_^*k*^	Difference of observed and image obtained leaf areas in group *G*_*k*_(*l*).
*e*_*mc*j_^*k*^	*a*_*oj*_^*k*^ − *a*_*mc*j_^*k*^	Difference of observed and Monte Carlo estimated leaf areas in group *G*_*k*_(*l*).

**Table 4:** Estimation errors for observed and image obtained variables averages and standard deviations.

Symbol	Formal expression	Description
*δ*_*l*_^*k*^	$\frac{\sum_{1}^{n_{k}}e_{lj}^{k}}{n_{k}}$	Leaf length average deviation in the group *G*_*k*_(*l*).

*δ*_*h*_^*k*^	$\frac{\sum_{1}^{n_{k}}e_{hj}^{k}}{n_{k}}$	Leaf width average deviation in the group *G*_*k*_(*l*).

*δ*_*a*_^*k*^	$\frac{\sum_{1}^{n_{k}}e_{aj}^{k}}{n_{k}}$	Leaf area average deviation in the group *G*_*k*_(*l*).

${\overset{-}{\delta }}_{l}$	$\frac{\sum_{1}^{n}\delta_{l}^{k}}{n}$	Leaf length average deviation in *C*_*G*_.

${\overset{-}{\delta }}_{h}$	$\frac{\sum_{1}^{n}\delta_{h}^{k}}{n}$	Leaf width average deviation in *C*_*G*_.

*σ*_*δl*_	${\left(\frac{\sum_{1}^{n_{k}}{\left(e_{lj}^{k}{-\delta }_{l}^{k}\right)}^{2}}{\left(n_{k}-1\right)}\right)}^{1/2}$	Standard deviation of *e*_*lj*_^*k*^.

*σ*_*δh*_	${\left(\frac{\sum_{1}^{n_{k}}{\left(e_{hj}^{k}-\delta_{h}^{k}\right)}^{2}}{\left(n_{k}-1\right)}\right)}^{1/2}$	Standard deviation of *e*_*hj*_^*k*^.

**Table 5:** Auxiliary statistics *λ*_*l*_, *λ*_*h*_, *θ*_*l*_, *θ*_*h*_, *λ*_*a*_, *β*_*a*_, *λ*_*amc*_ and *β*_*amc*_ used to obtain the set of leaves with estimation errors in range for a reliable estimation.

Symbol	Description	Reference equation
*λ*_*l*_	Proportion of leaves in *C*_*G*_ for which *δ*_*l*_^*k*^ satisfies: ${\overset{-}{\delta }}_{l}-\sigma_{\delta l}\;\leq \delta_{l}^{k}\leq {\overset{-}{\delta }}_{l}+\sigma_{\delta l}$	((E.5))
*λ*_*h*_	Proportion of leaves in *C*_*G*_ for which *δ*_*h*_^*k*^ satisfies: ${\overset{-}{\delta }}_{h}-\sigma_{\delta h}\leq \delta_{h}^{k}\leq {\overset{-}{\delta }}_{h}+\sigma_{\delta h}$	((E.6))
*θ*_*l*_	Proportion of leaves in *C*_*G*_ for which *δ*_*l*_^*k*^ do not satisfies: ${\overset{-}{\delta }}_{l}-\sigma_{\delta l}\;\leq \delta_{l}^{k}\leq {\overset{-}{\delta }}_{l}+\sigma_{\delta l}$	((E.7))
*θ*_*h*_	Proportion of leaves in *C*_*G*_ for which *δ*_*h*_^*k*^ do not satisfies: ${\overset{-}{\delta }}_{h}-\sigma_{\delta h}\leq \delta_{h}^{k}\leq {\overset{-}{\delta }}_{h}+\sigma_{\delta h}$	((E.8))
*λ*_*a*_	Proportion of leaves in *C*_*G*_ for which *δ*_*l*_^*k*^ and *δ*_*h*_^*k*^ satisfies: ${\overset{-}{\delta }}_{l}-\sigma_{\delta l}\;\leq \delta_{l}^{k}\leq {\overset{-}{\delta }}_{l}+\sigma_{\delta l}$ and ${\overset{-}{\delta }}_{h}-\sigma_{\delta h}\leq \delta_{h}^{k}\leq {\overset{-}{\delta }}_{h}+\sigma_{\delta h}$, and *e*_*aj*_^*k*^ ≥ 0.	((E.9))
*β*_*a*_	Proportion of leaves in *C*_*G*_ for which *δ*_*l*_^*k*^ and *δ*_*h*_^*k*^ do not satisfies: ${\overset{-}{\delta }}_{l}-\sigma_{\delta l}\;\leq \delta_{l}^{k}\leq {\overset{-}{\delta }}_{l}+\sigma_{\delta l}$ and ${\overset{-}{\delta }}_{h}-\sigma_{\delta h}\leq \delta_{h}^{k}\leq {\overset{-}{\delta }}_{h}+\sigma_{\delta h}$	((E.10))
*λ*_*amc*_	Proportion *λ*_*amc*_ equivalent to *λ*_*a*_ respectively but linked to leaf area estimation by Monte Carlo method (cf. (2)).	((E.11))
*β*_*amc*_	Proportion *β*_*amc*_ equivalent to *β*_*a*_ but linked to leaf area estimation by Monte Carlo method (cf. (2)).	((E.12))

**Table 6:** Numbers *n*_*k*_ of whole leaves classified in groups *G*_*k*_(*l*) formed by leaf sizes varying in corresponding length intervals *I*_*k*_.

*k*	*I*_*k*_	*n*_*k*_	*k*	*I*_*k*_	*n*_*k*_
1	[0, 10)	10	24	[230, 240)	24
2	[10, 20)	43	25	[240, 250)	23
3	[20, 30)	38	26	[250, 260)	15
4	[30, 40)	38	27	[260, 270)	21
5	[40, 50)	32	28	[270, 280)	16
6	[50, 60)	37	29	[280, 290)	12
7	[60, 70)	43	30	[290, 300)	10
8	[70, 80)	32	31	[300, 310)	9
9	[80, 90)	34	32	[310, 320)	9
10	[90, 100)	38	33	[320, 330)	4
11	[100, 110)	28	34	[330, 340)	7
12	[110, 120)	40	35	[340, 350)	3
13	[120, 130)	28	36	[350, 360)	4
14	[130, 140)	29	37	[360, 370)	3
15	[140, 150)	19	38	[370, 380)	3
16	[150, 160)	27	**39**	**[380, 390)**	**1**
17	[160, 170)	19	**40**	**[390, 400)**	**1**
18	[170, 180)	14	**41**	**[400, 410)**	**1**
19	[180, 190)	17	**42**	**[410, 420)**	**1**
20	[190, 200)	21	**43**	**[420, 430)**	**1**
21	[200, 210)	19	**44**	**[430, 440)**	**0**
22	[210, 220)	20	**45**	**[440, 450)**	**0**
23	[220, 230)	14	**46**	**[450, 460)**	**1**

**Table 7:** Direct comparison statistics for different ST(*x*) range values.

ST(*x*)	${\overset{-}{\delta }}_{h}$	*σ*_*δh*_	${\overset{-}{\delta }}_{l}$	*σ*_*δl*_	*θ*_*l*_	*θ*_*h*_	*λ*_*l*_	*λ*_*h*_
ST(68)	0.4493	0.2721	24.0157	23.6548	0.0161	0.1038	0.9839	0.8962
ST(128)	0.2599	0.2576	5.0342	13.7282	0.0049	0.0445	0.9951	0.9555
ST(192)	−0.1291	0.2496	3.8965	12.9700	0.0049	0.1669	0.9951	0.8331

**Table 8:** Proportions of overestimation and underestimation of leaf area and selection index values for a given ST(*x*) range.

ST(*x*)	*λ*_*a*_	*β*_*a*_	IS_*x*_	*λ*_*amc*_	*β*_*amc*_	IS_*xmc*_
ST(68)	0.6820	0.3180	0.4662	0.6666	0.3344	0.5016
ST(128)	0.7005	0.2995	0.4275	0.7197	0.2803	0.3894
ST(192)	0.4982	0.5018	1.0072	0.4917	0.5083	1.0337

**Table 9:** RMSD calculated by using observed versus image calculated variables.

ST(*x*)	RMSD(*h*_*o*_, *h*_*d*_)	RMSD(*l*_*o*_, *l*_*d*_)	RMSD(*a*_*o*_, *a*_*d*_)	RMSD(*a*_*o*_, *a*_*mc*_)
ST(68)	0.4590	26.4500	360.8746	151.2869
ST(128)	0.4016	12.9587	99.1725	90.6759
ST(192)	0.7303	10.8674	155.3371	160.6715

## Appendices

We now explain how to identify the interval of tolerance of similarity ST(*x*) that yields accurate estimations for the observed leaf area values. The task, is achieved through statistical methodologies, which requires the completion of the following steps.

### A. Grouping Leaf Data: *l*_*o*_ and *h*_*o*_

(A.1) Identify the maximum observed leaf length (*l*_max⁡_).
(A.2) Chose a positive integer *n* and define a partition of the interval [0, *l*_max⁡_] with norm *q* = (*l*_max⁡_/*n*).
(A.3) Form the collection ⋃_1_^*n*^(*I*_*k*_) of *n* disjoint intervals of the form *I*_*k*_ = [*q*(*k* − 1), *qk*) with 1 ≤ *k* ≤ *n*. This collection is denoted thought *P*_0_^*l*_max⁡_^.
(A.4) For each value of the index *k* identify the group *G*_*k*_(*l*) of leaves whose lengths are contained in *I*_*k*_. Notice that *G*_*k*_(*l*) holds the leaves whose size differences are bounded by *q*.
(A.5) For each value of the index *k* obtain and record *n*_*k*_ standing for the number of leaves in the group *G*_*k*_(*l*).
(A.6) For each value of the index *k* introduce an index *j* such that 1 ≤ *j* ≤ *n*_*k*_ and label as *l*_*oj*_^*k*^, *h*_*oj*_^*k*^ and *a*_*oj*_^*k*^ respectively, the straight length, width and area of the *j*th leaf in *G*_*k*_(*l*). The character *a*_*oj*_^*k*^ denotes the associated estimations of leaf area obtained by means of (1).
(A.7) Form and record the collection ⋃_1_^*n*^ *G*_*k*_(*l*) of all groups of leaves *G*_*k*_(*l*). This collection is denoted by means of *C*_*G*_.
(A.8) Obtain the average length ${\overset{-}{l}}_{o}^{k}$ for each group of leaves *G*_*k*_(*l*). That is, calculate and record
  (A.1) $$\begin{array}{c} {\overset{-}{l}}_{o}^{k}=\frac{1}{n_{k}}\overset{n_{k}}{\underset{1}{\sum }}l_{oj}^{k}. \end{array}$$
(A.9) Obtain the average width ${\overset{-}{h}}_{o}^{k}$ for each group of leaves *G*_*k*_(*l*). That is, calculate and record
  (A.2) $$\begin{array}{c} {\overset{-}{h}}_{o}^{k}=\frac{1}{n_{k}}\overset{n_{k}}{\underset{1}{\sum }}h_{oj}^{k}. \end{array}$$
(A.10) Obtain the average area ${\overset{-}{a}}_{o}^{k}$ for each group of leaves *G*_*k*_(*l*). That is, calculate and record,
  (A.3) $$\begin{array}{c} {\overset{-}{a}}_{o}^{k}=\frac{1}{n_{k}}\overset{n_{k}}{\underset{1}{\sum }}a_{oj}^{k}. \end{array}$$

### B. Obtaining Length, Width and Area from the Image of each Leaf

(B.1) For processing all digital images, we chose a specified color format with a number *C*_max⁡_ of colors.
(B.2) For processing the digital images of all collected leaves, we choose different intervals of tolerance of similarity ST(*x*) = [0, *x*], with the upper bound *x* satisfying 0 ≤ *x* ≤ *C*_max⁡_ − 1.
(B.3) For a picked ST(*x*) interval, for 1 ≤ *j* ≤ *n*_*k*_ use the algorithm (a) described in the method section to obtain *l*_*dj*_^*k*^ and *h*_*dj*_^*k*^, which respectively denote the length and width of the image of the *j*th leaf in *G*_*k*_(*l*). Also, obtain *a*_*dj*_^*k*^ and *a*_*mc**j*_^*k*^, which respectively stand for leaf area obtained from the image and calculated by means of (1) and (2) respectively. Record these values.
(B.4) interval obtain and record the concomitant averages ${\overset{-}{l}}_{d}^{k}$, ${\overset{-}{h}}_{d}^{k}$, ${\overset{-}{a}}_{d}^{k}$ and ${\overset{-}{a}}_{cm}^{k}$ (cf. (A.1) through (A.3)).

### C. Obtain Estimation Errors between the Observed and Image Obtained Values in Steps A and B

(C.1) For the picked ST(*x*) interval and for 1 ≤ *j* ≤ *n*_*k*_, calculate the leaf length approximation errors through
  (C.1) $$\begin{array}{c} e_{lj}^{k}=l_{oj}^{k}-\;l_{dj}^{k}. \end{array}$$
(C.2) For the picked ST(*x*) interval and for 1 ≤ *j* ≤ *n*_*k*_, calculate the individual leaf width approximation errors through
  (C.2) $$\begin{array}{c} e_{hj}^{k}=h_{oj}^{k}-h_{dj}^{k}. \end{array}$$
(C.3) For the picked ST(*x*) interval and for 1 ≤ *j* ≤ *n*_*k*_, calculate the leaf area approximation errors through
  (C.3) $$\begin{array}{c} e_{aj}^{k}=a_{oj}^{k}-a_{dj}^{k}. \end{array}$$
(C.4) For the picked ST(*x*) interval and for 1 ≤ *j* ≤ *n*_*k*_, calculate the leaf area approximation errors linked to the Monte Carlo method through
  (C.4) $$\begin{array}{c} e_{mcj}^{k}=a_{oj}^{k}-a_{mcj}^{k}. \end{array}$$

### D. Obtain the Average Deviations Produced by the Individual Estimation Errors

(D.1) For the picked ST(*x*) interval, obtain the average leaf length deviations *δ*_*l*_^*k*^, which are calculated by averaging the *e*_*lj*_^*k*^ values. That is,
  (D.1) $$\begin{array}{c} \delta_{l}^{k}=\frac{1}{n_{k}}\overset{n_{k}}{\underset{1}{\sum }}e_{lj}^{k}. \end{array}$$
We notice that $\delta_{l}^{k}=({\overset{-}{l}}_{o}^{k}-{\overset{-}{l}}_{d}^{k})$ and also that negative values of *δ*_*l*_^*k*^ imply that in a lot *G*_*k*_(*l*) most image assessments *l*_*dj*_^*k*^ overestimate observed *l*_*oj*_^*k*^ values.
(D.2) For the picked ST(*x*) interval, obtain the average leaf width deviations *δ*_*h*_^*k*^, which are calculated by averaging the *e*_*hj*_^*k*^ values. That is,
  (D.2) $$\begin{array}{c} \delta_{h}^{k}=\frac{1}{n_{k}}\overset{n_{k}}{\underset{1}{\sum }}e_{hj}^{k}. \end{array}$$
notice that since $\delta_{h}^{k}=\left({\overset{-}{h}}_{o}^{k}-{\overset{-}{h}}_{d}^{k}\right)$, negative values of *δ*_*h*_^*k*^ imply that in a group *G*_*k*_(*l*) most image assessments *h*_*dj*_^*k*^ overestimate observed *h*_*oj*_^*k*^ values.
(D.3) For the picked ST(*x*) interval, calculate the average leaf area deviations *δ*_*a*_^*k*^ by averaging the *e*_*aj*_^*k*^ values. That is,
  (D.3) $$\begin{array}{c} \delta_{a}^{k}=\frac{1}{n_{k}}\overset{n_{k}}{\underset{1}{\sum }}e_{aj}^{k}. \end{array}$$
again since $\delta_{a}^{k}={\overset{-}{a}}_{o}^{k}-{\overset{-}{a}}_{d}^{k}$ negative values of *δ*_*a*_^*k*^ imply that in a group *G*_*k*_(*l*) most image assessments *a*_*dj*_^*k*^ overestimate observed *a*_*oj*_^*k*^ values.
(D.4) For the picked ST(*x*) interval, calculate ${\overset{-}{\delta }}_{l}$, the average value of deviations *δ*_*l*_^*k*^ taken over *C*_*G*_. Calculate also the associated standard deviation *σ*_*δl*_.
(D.5) For the picked ST(*x*) interval, calculate ${\overset{-}{\delta }}_{h}$, the average value of deviation *δ*_*h*_^*k*^ taken over *C*_*G*_. Calculate also the associated standard deviation *σ*_*δh*_.

### E. Criteria for Selecting the *ST*(*x*) Interval That Produces the Highest Correspondence Level between Image Obtained Measurements and Those Obtained Directly from Collected Leaves

(E.1) For a given range of similarity values ST(*x*) = [0, *x*], identify the leaves satisfying the conditions
  (E.1) $$\begin{array}{c} {\overset{-}{\delta }}_{h}\geq 0, \end{array}$$
  (E.2) $$\begin{array}{c} {\overset{-}{\delta }}_{l}\geq 0, \end{array}$$
  (E.3) $$\begin{array}{c} {\overset{-}{\delta }}_{l}-\sigma_{\delta l}\leq \delta_{l}^{k}\leq {\overset{-}{\delta }}_{l}+\sigma_{\delta l}, \end{array}$$
  (E.4) $$\begin{array}{c} {\overset{-}{\delta }}_{h}-\sigma_{\delta h}\leq \delta_{h}^{k}\leq {\overset{-}{\delta }}_{h}+\sigma_{\delta h}. \end{array}$$
(E.2) Calculate the proportion *λ*_*l*_ of leaves in *C*_*G*_ that comply with the condition (E.3) through
  (E.5) λl=∑k=1n∑j=1nk[ldjk ∣ leaves  in  Gk  that  comply  with   condition  (E.3)]×(∑k=1n∑j=1nklojk)−1.
(E.3) Calculate the proportion *λ*_*h*_ of leaves in *C*_*G*_ that comply with the condition (E.4) through
  (E.6) λh=∑k=1n∑j=1nk[hdjk ∣ leaves  in  Gk  that  comply  with   condition  (E.4)]×(∑k=1n∑j=1nkhojk)−1.
(E.4) Calculate the proportion *θ*_*l*_ of leaves in *C*_*G*_ that do not comply with the condition (E.3) through,
  (E.7) $$\begin{array}{c} \theta_{l}=1-\lambda_{l}. \end{array}$$
(E.5) Calculate the proportion *θ*_*h*_ of leaves in *C*_*G*_ that do not comply with the condition (E.4) through,
  (E.8) $$\begin{array}{c} \theta_{h}=1-\lambda_{h}. \end{array}$$
(E.6) Obtain the concomitant proportions of leaves in *C*_*G*_ that provide consistent leaf area estimations by the proxy of (1)
  (E.9) λa=∑k=1n∑j=1nk[adjk ∣ leaves  in  Gk  that  comply  with   condition  (E.3),(E.4)  and  eajk≥0]   ×(∑k=1n∑j=1nkaojk)−1.
(E.7) Calculate the proportion of leaves in *C*_*G*_ for which image estimated blade length and width measurements overestimate leaf area calculated through (1)
  (E.10) $$\begin{array}{c} \beta_{a}=1-\lambda_{a}. \end{array}$$
(E.8) The proportions *λ*_*a**mc*_ and *β*_*a**mc*_ equivalent to *λ*_*a*_ and *β*_*a*_ respectively but linked to leaf area estimation by Monte Carlo method (cf. (2)) that is,
  (E.11) λamc=∑k=1n∑j=1nk[amcjk ∣ leaves  in  Gk  that  comply  with   condition  (E.3),(E.4)  and  emcjk≥0]   ×(∑k=1n∑j=1nkaojk)−1,
  (E.12) $$\begin{array}{c} \beta_{amc}=1-\lambda_{amc}. \end{array}$$

Conditions (E.1) and (E.2) grant bounded estimation errors, for *h* and *l* respectively. Moreover, the groups of leaves that also satisfy conditions (E.3) and (E.4) can be identified as those groups for which image *l*_*d*_ and *h*_*d*_ estimations are closer to directly obtained *l*_*o*_ and *h*_*o*_ measurements. Therefore, groups in *C*_*G*_, which do not comply with conditions (E.1)–(E.4), denote the set of leaves with estimation errors out of range for a reliable estimation.
